# Is poor sleep quality associated with poor neurocognitive outcome in cancer survivors? A systematic review

**DOI:** 10.1007/s11764-022-01213-z

**Published:** 2022-05-02

**Authors:** A. Josephine Drijver, Quirien Oort, René Otten, Jaap C. Reijneveld, Martin Klein

**Affiliations:** 1 Department of Neurology and Brain Tumor Center Amsterdam at Amsterdam UMC, Vrije Universiteit Amsterdam, Amsterdam, The Netherlands; 2 Medical Library, Amsterdam UMC, Vrije Universiteit Amsterdam, Amsterdam, The Netherlands; 3 Department of Neurology, Stichting Epilepsie Instellingen Nederland (SEIN), Heemstede, The Netherlands; 4 Department of Medical Psychology and Brain Tumor Center Amsterdam at Amsterdam UMC, Vrije Universiteit Amsterdam, Amsterdam, The Netherlands

**Keywords:** Neurocognitive functioning, Sleep, Cancer survivors, Quality of life

## Abstract

**Purpose:**

Cancer-related neurocognitive impairment and poor sleep are prevalent in cancer survivors and have a negative impact on their quality of life. This systematic review studies the association between sleep disturbance and neurocognitive functioning, as well as the potential positive effects of sleep interventions on neurocognitive functioning in cancer survivors. In addition, we aimed at determining the potential positive effects of sleep interventions on neurocognitive functioning in this population.

**Methods:**

Following PRISMA guidelines for reporting systematic reviews and meta-analyses, a comprehensive PubMed, Embase, PsycINFO, and CINAHL search was performed. Inclusion criteria were adult cancer survivors, self-reported or objective measures of neurocognitive functioning and sleep quality, or reports on the association between sleep and neurocognitive functioning.

**Results:**

Of the 4,547 records retrieved, 17 studies were retained for this review. Twelve studies were correlational, and five reported on interventions aimed at improving sleep quality. All studies that included self-reported neurocognitive functioning found that poorer sleep was associated with worse neurocognitive functioning. In four out of eight studies, poorer sleep was associated with objective neurocognitive impairment. Three out of five interventional studies showed neurocognitive functioning improved with improved sleep.

**Conclusions:**

While poor sleep in cancer survivors is associated with *self-reported* neurocognitive impairment, the association between poor sleep and *objective* neurocognitive impairment is less evident.

**Implications for Cancer Survivors:**

It is important that care providers are aware of the association between sleep and neurocognitive functioning and that improving sleep quality can be a way to decrease neurocognitive impairment in cancer survivors.

**Supplementary Information:**

The online version contains supplementary material available at 10.1007/s11764-022-01213-z.

## Introduction

Survival rates for cancer patients have improved substantially with advances in cancer treatments. While cancer and cancer treatments are associated with an increased risk of cancer-related cognitive impairment (CRCI), its incidence may differ depending on the disease stage. Up to 75% of cancer survivors report neurocognitive impairment after cancer treatment [1]. Neurocognitive impairment is associated with higher levels of anxiety, depression, fatigue, and difficulty in keeping up with work and social obligations [2] and results in a decreased quality of life [3, 4]. In recent years, research in this topic has increased, resulting in numerous studies investigating causes of and treatments for cancer-related neurocognitive impairment in cancer survivors [5].

Multiple factors are associated with the development of CRCI, such as tumour- and treatment-related, genetic, and sociodemographic factors [6]. While these might all be relevant in patient care, many of these factors are non-modifiable and therefore unfortunately not suitable as treatment targets. However, lifestyle, which includes factors such as exercise, diet, and sleep, is highly modifiable. In healthy and at-risk populations, research in this field is extensive and suggests that multi-domain interventions targeting several risk factors and mechanisms simultaneously might be necessary for an optimal preventive effect [7]. In the cancer field, however, research on lifestyle and neurocognitive function is limited, and the number of studies specifically investigating risk factors for developing CRCI and treatments of neurocognitive impairment is limited.

An unhealthy diet and insufficient physical activity have been identified as potential risk factors for neurocognitive impairment and thus are treatment targets for improving neurocognitive functioning in cancer survivors [8, 9]. While several cross-sectional studies in the aging population suggest that poor sleep might be associated with poor neurocognitive functioning [10, 11] as well as dementia [12, 13], studies on this association in cancer survivors are rare. In healthy subjects, a strong association has been found between self-reported and objective poor sleep quality, poor sleep hygiene, and insomnia on the one hand, and neurocognitive deficits on the others [14]. For example, sleep deprivation negatively impacts working memory, attention, long-term memory, and decision-making in healthy individuals [15, 16]. Also, when compared to subjects without sleep problems, subjects with insomnia show impaired episodic memory, working memory, and problem-solving abilities [17].

Poor sleep and insomnia are common in patients with breast cancer, prostate cancer, and lung and head and neck cancer and are reported by up to 80% of all cancer patients during treatment [18–21]. Consequently, these patients are at risk to develop neurocognitive deficits. Both hyper- and hyposomnia are associated with increased mortality [22, 23]. While some survivors may recover from these issues, a recent health survey demonstrated that 58% of cancer survivors continue to experience sleep problems after treatment [24, 25]. Not surprisingly, sleep issues are associated with other symptoms such as fatigue and a decreased quality of life and are often present in cancer patients and survivors who report psychological distress [26].

To ameliorate sleep disturbances in cancer patients, cognitive behavioural therapy (CBT) and movement interventions such as yoga and Qigong/Tai Chi have been studied [27, 28]. A meta-analysis found that any type of aerobic exercise could have a positive impact on sleep disturbances in cancer patients [28]. A recent review studying different treatments for sleep–wake disturbances in cancer concluded that CBT was preferred [29]. It is not known, however, whether CBT also is the preferred method for treating neurocognitive impairment resulting from poor sleep in cancer survivors.

The aim of this review is to investigate whether neurocognitive impairment is associated with poor sleep in cancer survivors: first, by providing an overview of studies investigating associations between neurocognitive functioning and sleep in cancer survivors and, second, by discussing intervention studies aimed at improving sleep in cancer survivors and the effect of these interventions on neurocognitive functioning. By establishing whether sleep disturbances are associated with neurocognitive impairment, it might be possible to identify patients who are at risk for developing neurocognitive dysfunction resulting from poor sleep. Discovering which type of sleep intervention is most impactful at improving neurocognitive functioning in cancer survivors will allow for recommendations regarding the preferred type of treatment for cancer survivors who report both neurocognitive impairments and sleep issues.

## Methods

### Electronical data sources and search

A literature search was performed by RO and AJD, based on the Preferred Reporting Items for Systematic Reviews and Meta-Analysis (PRISMA)-statement (www.prisma-statement.org). To identify all relevant publications about the relation between sleep and neurocognitive decline in cancer patients, we performed systematic searches in the bibliographic databases PubMed, Embase.com, PsycINFO (via EBSCO), and CINAHL (via EBSCO) from all years till 2021 May 26th. Search terms included controlled terms (MeSH in PubMed and Emtree in Embase, etc.) as well as free text terms. Search terms expressing ‘cancer’ were used in combination with search terms comprising ‘cognition’ and search terms comprising ‘sleep’.

The references of the identified manuscripts were searched for relevant publications. The full search strategies for all databases can be found in the supplementary information.

### Inclusion and exclusion criteria

Inclusion criteria for the manuscripts were (1) cancer patients who have completed treatment, except for hormonal treatments; (2) self-reported sleep (sr-Sleep) or objective sleep (oSleep) assessments; (3) self-reported neurocognitive functioning (sr-NCF) or objective neurocognitive functioning (oNCF); (4) data of an association between sleep and neurocognitive functioning; (5) the manuscript was in English; and (6) the manuscript presented original peer-reviewed research. The following exclusion criteria were used: (1) patients undergoing or anticipated to be undergoing primary cancer treatments (surgery, chemotherapy, radiotherapy, or immunotherapy); (2) animal research; (3) paediatric population; and (4) case reports, reviews, study protocols and conference abstracts.

### Study selection, data extraction, and quality assessment

After removal of all duplicates, AJD first removed all manuscripts that could be excluded based on title and abstract. Next, AJD and QO reviewed the remaining full text manuscripts. The manuscripts that fulfilled the criteria were included in the review. Disagreements were discussed, until agreement was reached regarding inclusion. The included manuscripts were divided into either cross-sectional or intervention studies.

Data extraction and quality assessment were performed by AJD and QO. The following data was extracted from the manuscripts: author, year of publication, population, design, sleep measurements, neurocognitive measurements, intervention when applicable, and key findings.

The Appraisal tool for Cross-Sectional Studies (AXIS) was used to assess the quality of the cross-sectional studies [30]. The aim of the tool is to aid systematic interpretation of a cross-sectional study and to inform decisions about the quality of the study.

To assess the quality in the interventional studies, the Cochrane risk-of-bias tool for randomized trials version 2 (RoB 2) was used [31]. RoB 2 is structured into a fixed set of domains of bias, focusing on different aspects of trial design, conduct, and reporting. Judgement about the risk of bias arising from each domain can be 'Low' or 'High' risk of bias or can express 'Some concerns'.

## Results

### Inclusion of studies

**Fig. 1:**
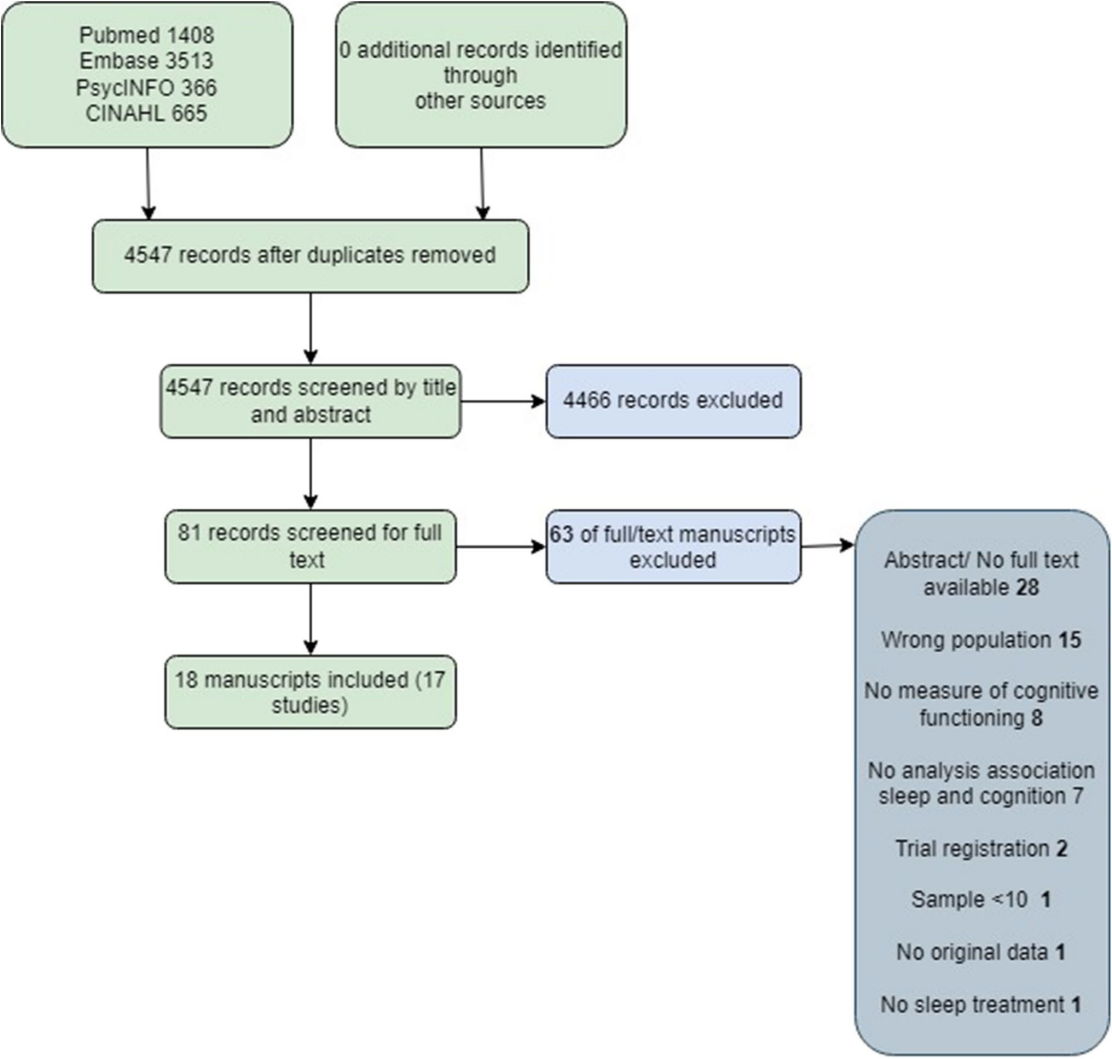
Flowchart of included manuscripts

The search resulted in 4,547 records after removal of duplicates (Fig. 1). After screening of titles and abstracts, 81 records remained for full-text analysis. Of the 81 records, 18 were included after the full-text review. Examination of the reference lists of these 18 manuscripts did not identify additional papers. Two manuscripts by Henneghan et al. [32, 33] were considered as a single study since the same patient sample was reported in both papers, resulting in a total number of 17 studies included in this review.

### Characteristics of the included studies

Of the 17 studies included in the final review, 12 were observational and 5 were interventional (Tables 1 and 2).

**Table 1:** Included cross-sectional studies

First author, year and journal	Design	Participants	Sleep measure	Measure objective NCF	Measure self-reported NCF	Key findings
Caplette-Gingras 2013[34] *BEHAVIORAL SLEEP MEDICINE*	Cross-sectional	67 breast cancer patients after treatment completion	ISI Daily sleep diary	Multi-domain neuropsychological test battery	CFQ ASS PRS	*oNCF:* Patients with insomnia performed worse on verbal episodic memory compared to good sleepers F(1,16) = 9.19, *p* = 0.004, *d* = 0.88 *sr-NCF:* Patients with insomnia showed lower scores on the ASS, *F* (1,16) = 8.49, *p* = 0.005, *d* = 0.84
Ehlers 2018 [35] *BMC CANCER*	Cross-sectional	271 breast cancer patients who had completed treatment	Accelerometer and diary	BrainBaseline (mobile app): Task-Switch and Trail Making tests	-	*oNCF:* Sleep was not associated with performance on the NP tests, *p* > 0.05
Ehlers 2020 [36] *CANCER MEDICINE*	Prospective	380 breast cancer survivors	PSQI	-	FOF	*sr-NCF:* There was a direct association between sleep disturbance and memory impairment on baseline (*z* = − 4.02) *p* < 0.001), and at 6 months follow-up (*z* = − 2.04, *p* = 0.02)
Hartman 2015 [37] *PSYCHO-ONCOLOGY*	Cross-sectional	136 breast cancer survivors	Question: ‘On average, how many hours of sleep do you get per night?’	Mindstreams (computerized neuropsychological test battery)	-	*oNCF:* More hours of sleep were associated with better verbal functioning (*β* = 2.69, SE = 0.98, *p* = 0.007) and decreased odds of impairment (OR = 0.52, 95% CI: 0.33–0.82)
Henneghan, Carter 2018 [33]^a^ *J CANCER SURVIVORSHIP*	Cross-sectional	90 breast cancer survivors, 6 months to 10 years after treatment	PSQI ESS	HVLT-R COWAT TMT A + B	FACT-Cog	*oNCF:* Sleep was not associated with objective neurocognitive functioning (*p* > 0.05) *sr-NCF:* Perceived cognitive impairment scale was moderately correlated with sleep quality subscales (*r* = − .38 to − .49, *p* < 0.001)
Henneghan, Stuifbergen 2018 [32]^a^ *PSYCHO-ONCOLOGY*	Cross-sectional	90 breast cancer survivors, 6 months to 10 years after treatment	PSQI ESS		FACT-Cog	*sr-NCF:* Sleep quality was correlated with overall FACT-Cog score (*r* = − .54), perceived cognitive impairment (r = − 0.51) and perceived cognitive abilities (*r* = − 0.46, *p* < 0.05)
Hutchinson 2021 [38] *EUR J CANCER CARE*	Cross-sectional	30 hematological cancer patients, 1–5 years after allogeneic SCT	PSQI	-	FACT-Cog	*sr-NCF:* better sleep quality was associated with better perceived cognitive abilities (*r* = − 0.46, *p* < 0.01), but not with perceived cognitive impairment
Jean-Pierre 2015 [39] *SLEEP MEDICINE*	National health survey data	164 cancer survivors	Diagnosis sleep disorders, questions about sleep quality	-	Question: ‘Are you limited in any way because of difficulty remembering or because you experience periods of confusion?’	*sr-NCF:* Cancer survivors with insomnia were more likely to report memory problems than cancer survivors without insomnia (OR = 13.18, 95% CI: 1.31–132.93, *p* < 0.03)
Jung 2017 [40] *SUPPORT CANCER CARE*	Cross-sectional	180 thyroid cancer patients after thyroidectomy	PSQI	Digit Span test COWAT	AFI	*oNCF:* no significant correlation between sleep quality and oNCF *sr-NCF:* Score on the AFI was correlated with sleep problems (*r* = − 0.39*, p* < 0.001)
Liou 2019 [41] *JNCI CANCER SPECTRUM*	Cross-sectional	1072 breast cancer survivors	ISI	-	BCPT cognitive subscale	*sr-NCF:* BCPT cognitive score was significantly worse for mild (*β* = 0.49), moderate(*β* = 0.78), and severe (*β* = 1.58) insomnia compared to no insomnia, *p* < 0.01
SYARIF 2019 [42] *ASIAN/PACIFIC ISLAND NURSING JOURNAL*	Cross-sectional	163 breast cancer survivors and 80 controls	PSQI	HVLT	-	*oNCF:* Sleep quality explained part of the worse performance in verbal learning and memory of survivors compared to controls (*B* = − 2.26, *p* < 0.001)
Van DYK 2018 [43] *NPJ BREAST CANCER*	Cross-sectional	198 breast cancer patients who had completed primary treatment in the past 3 months	PSQI	Multi-domain neuropsychological test battery	-	*oNCF:* Sleep quality was correlated with multiple neurocognitive domains: Learning (*r* = − 0.18, *p* = .02), memory (*r* = -0.22, *p* < 0.01), attention (*r* = − 0.29, *p* < 0.01), and executive function (*r* = − 0.17, *p* = 0.02)
Von AH 2015 [44] *J OF PAIN AND SYMPTOM MANAGEMENT*	Cross-sectional	88 breast cancer survivors	PSQI	Multi-domain neuropsychological test battery	FACT-Cog	*oNCF:* Performance on the neuropsychological tests was not associated with sleep problems (*p* > 0.05) *sr-NCF:* Perceived cognitive impairment scale was significantly correlated with sleep problems (*r* = -0.29, *p* < 0.01)

*Abbreviations: ISI* Insomnia Severity Index, *CFQ* Cognitive Failures Questionnaire, *ASS* Actual State Scale, *PRS* Performance Rating Scale, *FACT-Cog* Functional Assessment of Cancer Therapy-Cognitive Function, *PSQI* Pittsburgh Sleep Quality Index, *FOF* Frequency of Forgetting scale, *ESS* Epworth Sleepiness Scale, *MMSE* Mini Mental State Examination, *CNT* Computerized Neuropsychological Test, *GSDS* General Sleep Disturbance Scale, *AFI* Attentional Function Index, *EORTC QLQ-C30* European Organisation for Research and Treatment for Cancer Quality of Life Questionnaire, *MOS* Medical Outcome Scale, *BCPT* Breast Cancer Prevention Trail, *BOMC* Blessed Orientation-Memory-Concentration, *MoCA* Montreal Cognitive Assessment, *HVLT* Hopkins Verbal Learning Test

^a^Same patient sample

**Table 2:** Included sleep intervention studies

First author and year	Design	Participants	Sleep measure	Measure objective NCF	Measure self-reported NCF	Intervention	Key findings
Gregoire 2020 [45] *PSYCHO-ONCOLOGY*	RCT	95 cancer survivors more than a year after active treatment completion	ISI	-	FACT-Cog	Group intervention consisting of self-care and self-hypnosis vs waitlist control	*Sleep*: sleep difficulties decreased (*d* = 0.58 *p* < 0.001) in the group intervention *sr-NCF:* sr-NCF improved in the intervention group (perceived cognitive impairment: *d* = − 0.43, *p* = 0.020; perceived cognitive abilities: *d* = − 0.51, *p* = 0.004)
Janelsins 2016 [46] *INTEGRATIVE CANCER THERAPIES*	RCT	328 cancer survivors	PSQI	-	MDASI memory item	Yoga program aimed at improving sleep vs control	*sr-NCF:* sr-NCI decreased more in the yoga group (-0.60) compared to the control group (− 0.16), *p* < 0.05
**Liou 2020 **[47] ***CANCER***	RCT	99 patients who completed active treatment at least 1 month prior to the study	ISI	Buschke Selective Reminding Test	Brown Attention-Deficit Disorder Scale	Acupuncture vs CBT-I	*oNCF:* Acupuncture group showed improvement on all trials of the BSRT while the CBT-I group only improved on one trial, there was no significant difference between the two groups (*p* > 0.05) *sr-NCF:* both acupuncture (*D* = 0.54 and 0.65) and CBT-I (*D* = 0.73 and 0.84) showed improvement at 8 and 20 weeks on the BADDS (*p* < 0.001). There was no significant difference between the two groups (*p* = 0.28)
Matthews 2014 [44] *ONCOLOGY NURSING FORUM*	RCT	56 BC survivors with chronic insomnia	Sleep diary ISI	-	AFI	CBT-I vs behavioural placebo treatment	*Sleep:* multiple sleep measures improved in the CBT-I group *sr-NCF:* The CBT-I group showed a small non-significant improvement compared to the control group on attentional function, *d* = 0.56, *p* = .07
Simeit 2004 [48] *SUPPORT CANCER CARE*	N-RCT	158 patients with various malignancies	PSQI	-	EORTC QLQ-C30 cognitive scale	Progressive muscle relaxation vs autogenic training vs control	*Sleep:* both intervention groups improved on sleep quality *Sr-NCF:* All groups improved over time on the cognitive functioning sub-scale, *F* = 14.1, *p* < 0.001. There was no significant difference between groups

*Abbreviations: MDASI* MD Anderson Symptom Inventory, *CBT-I* Cognitive Behavioural Therapy for Insomnia

The majority of included studies focused on patients with a single cancer type, being breast cancer (*n* = 10), thyroid cancer (*n* = 1), or haematological cancer (*n* = 2). Five studies consisted of a sample of patients with multiple cancer types (i.e., breast, prostate, lung, urogenital, gastrointestinal, haematological). Time since treatment completion ranged from 1 month to 5 years.

#### Neurocognitive evaluation: self-reported and objective measures

Five sleep-related studies included both objective measures and self-reports of neurocognitive functioning. Eight studies only used self-reports. The remaining four studies only reported objective neurocognitive functioning. The test batteries consisted of up to 13 tests, spanning domains such as verbal learning and memory, working memory, flexibility, and visuospatial functioning. Verbal learning tests, the Trail Making Test, and the Controlled Oral Word Association test were most prevalent.

The self-reports of neurocognitive functioning used were very heterogeneous. The Functional Assessment of Cancer Therapy-Cognitive Function (FACT-Cog, *n* = 4) and Attentional Function Index (AFI, *n* = 2) questionnaires were used most often. Both questionnaires are specifically developed for use in cancer patients with potential self-reported neurocognitive impairment related to chemotherapy use. The FACT-Cog indexes self-reported neurocognitive functioning in cancer patients in 4 subscales: perceived cognitive impairment, impact on quality of life, comments from others, and cognitive abilities perceived by others [49]. The AFI is a questionnaire that focuses on problems associated with working memory and attention in the performance of daily tasks [50].

#### Sleep evaluation: self-reported and objective measures

The majority of the 17 studies used self-reported ratings to capture sleep quality. Of these instruments the Pittsburgh Sleep Quality Index (PSQI) [51] was most often used (*n* = 9). The PSQI consists of 19 questions and produces seven component scores: subjective sleep quality, time until sleep onset, sleep duration, habitual sleep efficiency (time in bed relative to time asleep), sleep disturbances, use of sleep medication and daytime dysfunction, and a total score, where a higher score indicates worse sleep quality. Another frequently used questionnaire was the Insomnia Severity Index (*n* = 5) [52]. While most studies included a specific questionnaire as a measure of sleep, some used only one or two questions regarding sleep quality [37, 39]. A few manuscripts used sleep diaries [34, 35, 44], while only one study used an accelerometer as an objective measure of sleep quality [35].

### Cognitive functioning and sleep

#### Association between sleep and objective neurocognitive functioning

**Fig. 2:**
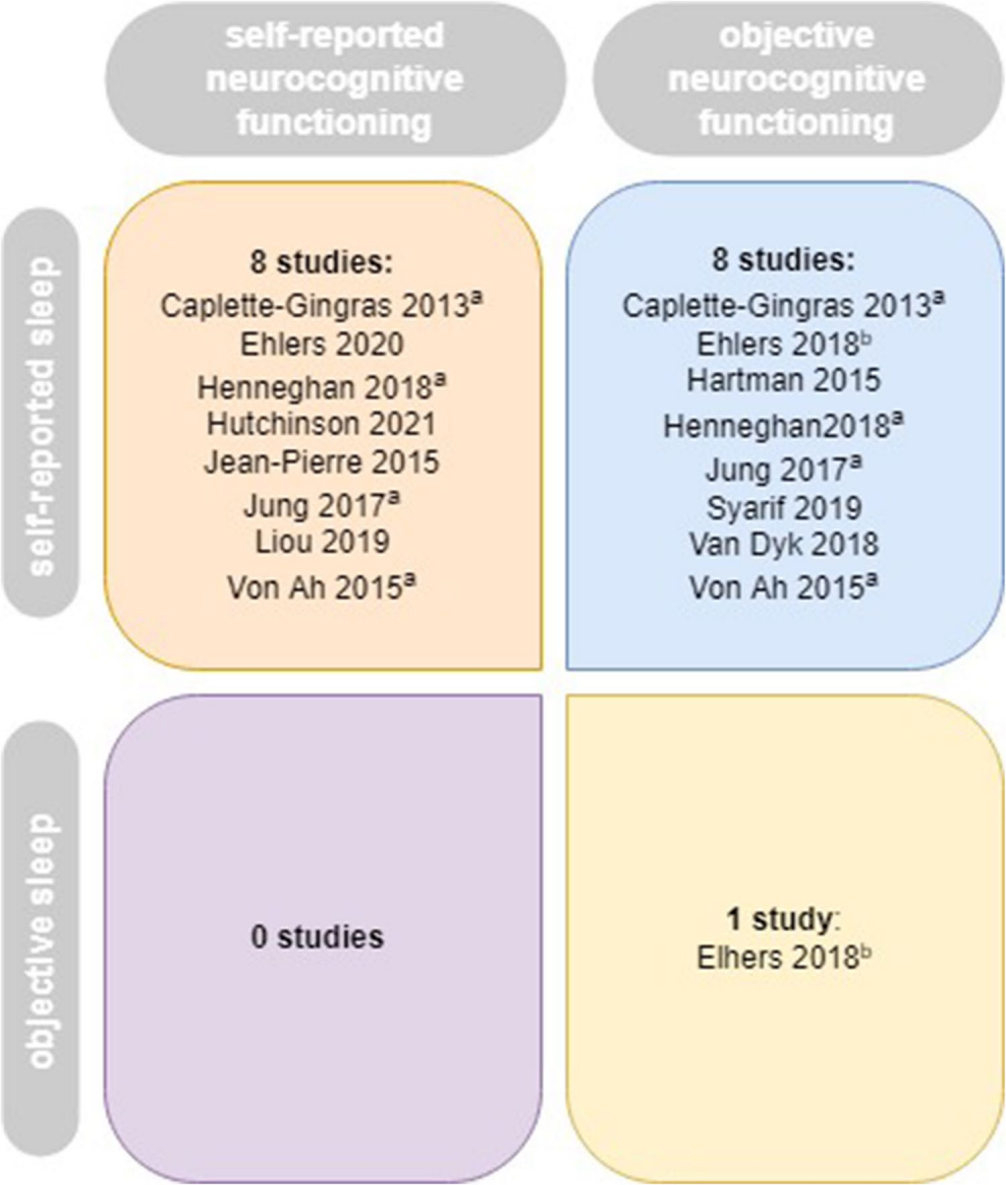
Overview of the measures used in the included cross-sectional studies: ^a^study included measures of both self-reported and objective neurocognitive functioning, ^b^study included measures of both self-reported and objective sleep

Most studies used the total scores of the PSQI or ISI questionnaires as a measure of sleep. Eight studies investigated the association between sleep and objective neurocognitive functioning, of which four found no association [33, 40, 53]. For an overview of the studies, see Fig. 2.

Of the eight studies, one study compared breast cancer survivors with and without insomnia on oNCF [34]. Patients with insomnia performed worse on verbal episodic memory (*p* = 0.004), with mostly moderate to large effect sizes for the individual verbal episodic memory tests (*d* = 0.51–0.89). There were no significant differences between the insomnia group and good sleepers on the domains of visual episodic memory, and attention and processing speed, nor executive functioning.

Four studies [33, 40, 43, 53] used correlational analyses to establish the association between oNCF and sleep. All four of them used the PSQI as the measure of sleep quality. Importantly, significant positive correlations between score on the PSQI global score and oNCF were found in one of those 4 studies, indicating that worse sleep quality was associated with worse oNCF [43]. In this study, sleep quality is correlated with domains of learning (*r* =  − 0.18, *p* = 0.02), memory (*r* =  − 0.22, *p* < 0.01), attention (*r* =  − 0.29, *p* < 0.01), and executive function (*r* =  − 0.17, *p* = 0.02), indicating moderate associations. Correlations were not significant for the visuospatial- and processing speed domains. The other three studies found no significant correlations between scores on the PSQI and oNCF [33, 40, 53].

Two of the eight studies reported the effect of sleep duration on oNCF [35, 37]. The study by Ehlers primarily focused on the effect of replacing 30 min of sedentary time with 30 min of sleep or physical activity in breast cancer survivors. Sleep was measured using an accelerometer and sleep diary, while the BrainBaseline, a mobile app with neuropsychological tests, was used to measure oNCF. The replacement of sedentary time was not significantly associated with oNCF (*p* > 0.05). The Hartman study showed that more reported hours spent asleep were associated with better oNCF [37]. Specifically, more hours of sleep per night were associated with better verbal functioning (*β* = 2.69, SE = 0.98, *p* = 0.007) and significantly lower odds of impairment on the verbal functioning domain (OR = 0.52). Syariff and colleagues analysed the predictive value of sleep quality, measured with the PSQI, on verbal learning and memory using multiple logistic regression [42]. Results showed that sleep quality partially explained differences in verbal learning and memory function between breast cancer survivors and healthy controls (*B* =  − 2.26, *p* < 0.001). However, one-third of the sample consisted of non-cancer female patients and no separate analysis for the cancer survivors was performed.

#### Association between sleep and self-reported neurocognitive functioning

**Fig. 3:**
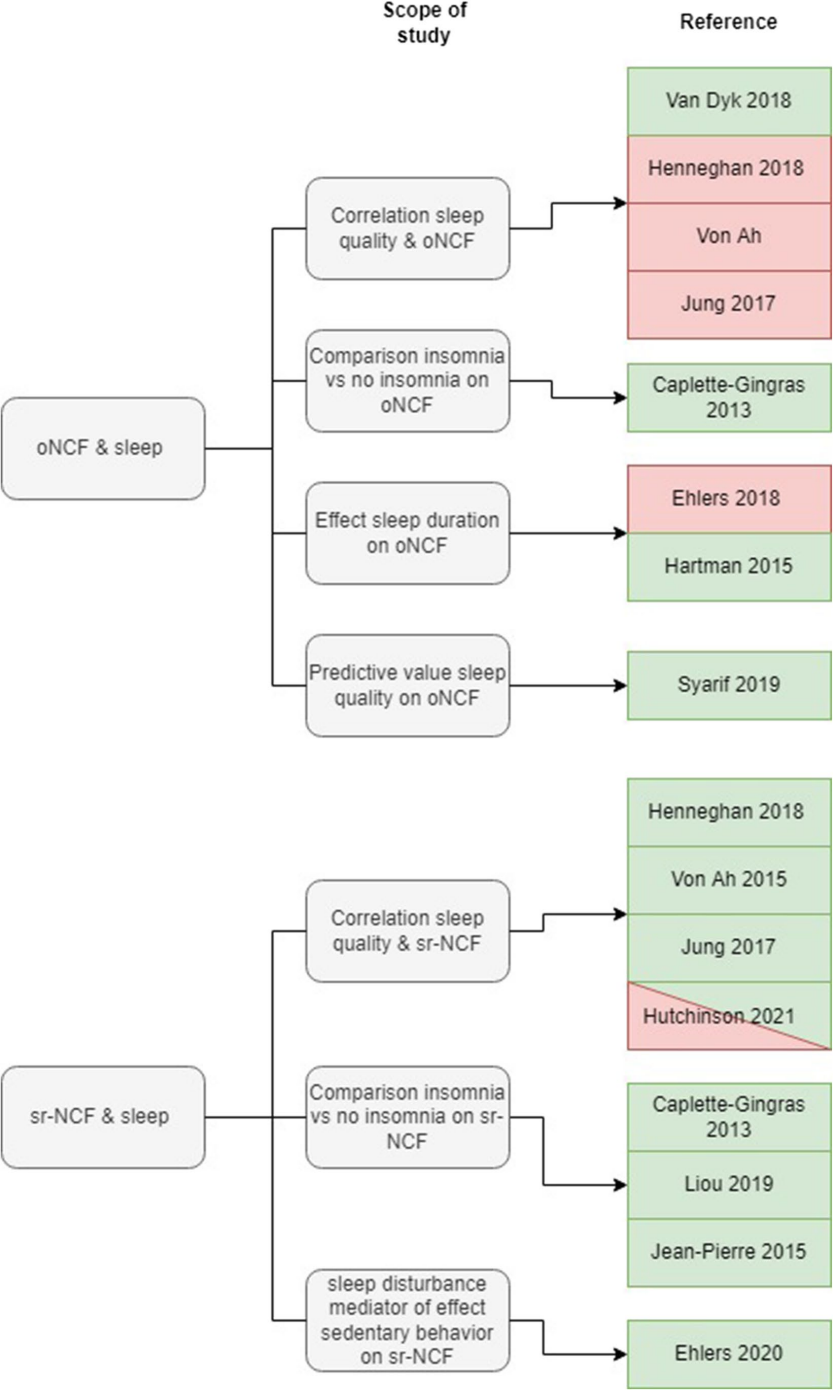
Overview of the studies investigating the association between objective neurocognitive functioning and sleep and self-reported neurocognitive functioning and sleep. References in green found a significant association; references in red found no significant association

Self-reported neurocognitive functioning was an outcome measure in 8 of the 13 studies listed in Table 1 (see Fig. 3). Seven studies found that sleep problems were associated with worse sr-NCF [32, 34, 36, 39–41, 53].

Four studies reported significant correlations between sleep quality and sr-NCF, indicating worse sleep quality was associated with increased self-reported neurocognitive impairment or better sleep quality was associated with improved sr-NCF [33, 38, 40, 53]. All studies used the PSQI as the measure for sleep quality. Three of these studies used the FACT-Cog as a measure of sr-NCF. In the first one, by Henneghan, moderate correlations were found between the FACT-Cog perceived cognitive impairment (PCI) subscale and 4 PSQI subscales (daytime dysfunction, efficiency, latency, and disturbance), with correlations varying between − 0.37 and − 0.49. The PSQI subscales sleep duration, sleep quality, and sleep medication use were not associated with the FACT-Cog PCI subscale [33]. In further analysis, the total PSQI score correlated significantly with the FACT-Cog total score as well as the FACT-Cog PCI and perceived cognitive abilities (PCA) subscales (*p* < 0.05) [32]. The study by Von Ah, also found a correlation between score on the FACT-Cog PCI scale and the total PSQI score (*r* =  − 0.29, *p* < 0.01). Sleep was not significantly correlated with perceived cognitive abilities. Hutchinson and colleagues did find a significant correlation between sleep quality and the PCA subscale in haematological cancer survivors 5 years after stem cell transplantation (*r* =  − 0.54, *p* < 0.01*)*, indicating better sleep quality was associated with improved sr-NCF [38]. They found no association between the PCI subscale and sleep quality in this population. The last correlational study by Jung and Visovatti used the Attentional Function Index (AFI) to measure sr-NCF [40]. Poorer sleep quality as reported by the PSQI total score was significantly correlated with poorer self-reported working memory and attentional functioning in everyday life, indexed by the AFI (*r* =  − 0.39, *p* < 0.001).

Ehlers et al. studied the effect of sedentary behaviour on sr-NCF and how sleep disturbance mediated this association in breast cancer survivors [36]. A direct association between sleep quality (PSQI) and sr-NCF (Frequency of Forgetting scale) was found (*z* =  − 4.02, *p* < 0.001 and *z* =  − 3.07, *p* = 0.001), showing that increased sleep disturbance was associated with increased self-reported neurocognitive impairment.

Three of the eight studies compared cancer patients with and without insomnia regarding their sr-NCF. Patients with insomnia scored lower on sr-NCF measures and were more likely to report memory problems than patients without insomnia [34, 39, 41]. Caplette-Gingras et al. used three measures of sr-NCF; the Cognitive Failures Questionnaire, the Actual State Scale, and the Performance Rating Scale [34]. They found survivors with insomnia to score lower on the ASS, indicating more neurocognitive problems (*p* = 0.005, *d* = 0.84), but not on the other two outcome measures. Liou et al. described sr-NCF for different levels of insomnia, measured with the Insomnia Severity Index (ISI) [41]. Scores on the Breast Cancer Prevention Trial cognitive subscale differed significantly between mild, moderate, and severe insomnia (*p* < 0.001), with patients with severe insomnia most likely to report lower sr-NCF (*β* = 1.58,* p* < 0.01). In another study, breast cancer survivors with insomnia were more likely to answer yes to the question ‘Are you limited in any way because of difficulty remembering or because you experience periods of confusion?’ than breast cancer survivors without insomnia (OR = 13.18, 95% CI: 1.31–132.93, *p* < 0.03) [39].

#### Effect of sleep interventions on neurocognitive functioning

Five studies that investigated sleep disorder treatments reported neurocognitive functioning as one of the outcome measures, two on sr-NCF, and three on oNCF.

Two studies investigated the effectiveness of Cognitive Behavioural Therapy for Insomnia (CBT-I). In the first study, 56 breast cancer survivors with chronic insomnia were randomized between the CBT-I treatment and a behavioural placebo control group [44]. The CBT-I group reported significant improvement in sleep latency, sleep efficiency, and total sleep time, when compared to the placebo control group. Importantly, the CBT-I group showed a small near-significant improvement in attentional function compared to the control group (*d* = 0.56, *p* = 0.07).

The second study compared CBT-I to acupuncture in cancer survivors who met the DSM-5 criteria for insomnia disorder [47]. Participants were randomized between an 8-week CBT-I treatment and 8 weeks of acupuncture. In both groups the score on the ISI improved significantly, indicating that symptoms of insomnia decreased. Both treatment groups showed an improvement on a verbal learning task, with small to moderate effect sizes (*D* = 0.25–0.50). Differences between the groups were non-significant. Both groups showed improvement of sr-NCF at 8 and 20 weeks, with moderate to large effect sizes (*D* = 0.54–0.84). There were no significant differences between groups.

Another study compared a group who underwent intervention consisting of self-care and self-hypnosis with a waiting list control group who received treatment 4 months later [45]. Participants in the experimental group showed a reduction in sleep difficulties (*d* = 0.58, *p* < 0.001) and also a decrease in sr-NCI (FACT-Cog) with an effect size of − 0.43,* p* = 0.02, while participants in the control group showed no difference between baseline and 4-month follow-up.

The final two studies applied physical interventions aimed at improving sleep. Janelsins and colleagues studied the effectiveness of a yoga program in improving sleep, measured with the PSQI [46]. The yoga program consisted of eight 75-min sessions over 4 weeks. Three-hundred-and-twenty-eight patients were randomized between the yoga program and a standard care control group. A previous paper showed that patients who participated in the yoga program demonstrated greater improvements in sleep quality compared to patients who received standard care [54]. The yoga group showed a greater decrease in sr-NCI, i.e., memory function (-0.60) compared to the control group (− 0.16), *p* < 0.05 [46].

A three-arm trial by Simeit including 229 patients with breast, kidney, or prostate cancer compared two different relaxation interventions; progressive muscle relaxation and autogenic training (i.e., relaxation using visualizations), against a control group that received a standard rehabilitation program [48]. Self-reported sleep, measured by the PSQI, improved in both intervention groups. All three groups showed a small improvement of sr-NCF, *F* = 14.1, *p* < 0.001, indicating the absence of an additional effect of both interventions on top of standard rehabilitation on sr-NCF.

### Quality and risk of bias assessment

**Fig. 4:**
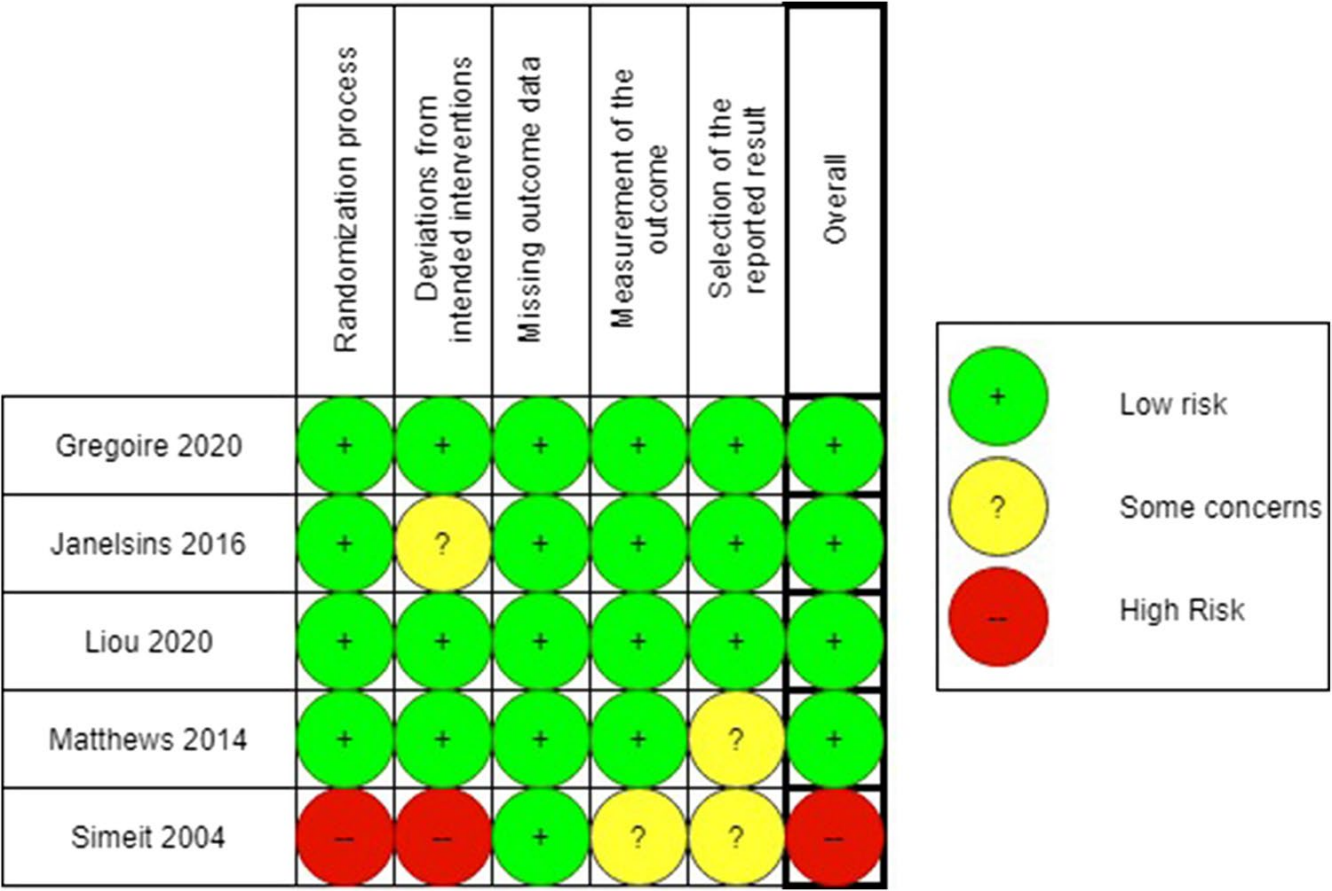
Risk of bias assessment for the (randomized) controlled trials

Using the Cochrane RoB 2 [31], a summary of the risk of bias of the 5 interventional studies is presented in Fig. 4. Four of the studies were at a low risk of bias, while one was at a high risk.

For the other 12 studies the AXIS tool was used to asses risk of bias, see Fig. 3. Overall, the quality of the papers was moderate to good. The most common issue was the failure to report on a justification for the sample size (*N* = 12) and on response rates and non-response bias (*N* = 11). For a detailed description of the AXIS tool and scores, see the supplementary information.

## Discussion

This review aimed at examining the association between sleep and neurocognitive functioning in cancer survivors. Multiple factors contribute to the development of sleep disturbances in cancer patients and survivors, such as neurotoxicity of the treatment, pain, and psychological symptoms [4, 55]. These factors are also associated with neurocognitive impairment, implying a possible relationship between sleep and neurocognitive functioning in cancer survivors as well. This is also in line with a survey among cancer survivors, where chemotherapy was strongly associated with lower self-reported neurocognitive functioning and more self-reported sleep difficulties [1].

We found limited evidence that in cancer survivors, sleep quality and quantity are associated with objective neurocognitive functioning (oNCF). Verbal memory/functioning seemed strongest associated with sleep disturbances, with one study suggesting that increased sleep duration is associated with better verbal functioning [37] and another study concluding that cancer survivors with insomnia performed worse on verbal episodic memory when compared to survivors who were good sleepers [34]. One out of three studies investigating the correlation between PSQI score and neuropsychological test performance found a significant correlation with the domains of learning, memory, attention, and executive function [43]. It is not clear why these correlations were not found in the other two studies, but an explanation might be that these studies used a less extensive test battery than the study by Van Dyk [43]. Also, these studies used the separate standardized test scores as a measure of oNCF instead of domain scores. In other populations (self-reported), sleep quality is also associated with oNCF; although similar to cancer research, there is great variability in the tests that are used [56, 57]. One recent study found that in memory clinic patients, only objective sleep measurements, and not self-reported, were associated with oNCF [58]. Unfortunately, only one of the included studies used an accelerometer, and this study found no association between oNCF and sleep [35].

Overall, there was heterogeneity of the neuropsychological tests used in the different papers, making it difficult to compare outcome measures. Furthermore, some studies used just a small number of tests, while others used an elaborate test battery. If only specific neurocognitive domains are impacted by sleep disturbances, a summarized score might not be sensitive enough. Further research is necessary to be able to confirm the association with the domains reported in this review.

All studies with self-reported neurocognitive functioning (sr-NCF) as an outcome measure were evidential for a positive association between sleep disturbances and self-reported neurocognitive impairment. Studies comparing cancer survivors with and without insomnia found evidence that insomnia was associated with poorer sr-NCF [34, 39, 41]. It is not possible to identify which other sleep-related factors are specifically associated with sr-NCF, since some studies used total scores of the PSQI, while others also reported subscales. Overall, the findings suggest that improving sleep quality could be a way to decrease sr-NCF.

The finding that poor sleep seems mostly associated with self-reported and not objective neurocognitive impairment might indicate that instead of causing neurocognitive impairment, sleep is associated with the impression that a patient has neurocognitive impairment. This is similar to findings in other patient groups and healthy populations [59, 60]. Previous research has shown that sr-NCF is also associated with symptoms of anxiety depression, quality of life, and fatigue [61, 62]. It has been shown that sleep and sr-NCF are part of a psychological symptom cluster in oncological patients [63]. It is likely that poor sleep increases fatigue and sr-NCF, while symptoms of anxiety and depression can lead to poorer sleep. This would also suggest that interventions aimed at improving sleep could benefit the whole symptom cluster.

The studies included in this review suggest that sleep interventions might indeed improve sr-NCF, but more research is needed. One study that found an effect of treatments targeting sleep problems used the self-reported neurocognitive functioning scale of the EORTC QLQ-C30 as an outcome measure. This scale consists of (only) two questions, one regarding memory and one regarding concentration. Although the improvement on this scale is encouraging, it would still be important to study the effect of sleep interventions on sr-NCF with more extensive outcome measures. Furthermore, the interventions in all five studies were all very different. More research is necessary before we can conclude whether these interventions could be effective in decreasing sr-NCF. Future interventional sleep studies are recommended to include at least a measure of sr-NCF.

To make future studies more comparable, it is recommended that they use similar measures for NCF and sleep. To measure oNCF, it is generally recommended to use the test battery from the International Cognition and Cancer Task Force, potentially supplemented by other tests [64]. The tests in this battery have been specifically selected for their ability to measure oNCF in cancer. However, since most studies found no association between sleep and performance on these tests, it is important to continue research into which tests could be more sensitive to detect possible oNCF changes associated with sleep in cancer survivors at all.

In terms of measuring sr-NCF, the neurocognitive functioning scale of the EORTC QLQ-C30 is sufficient to indicate whether a patient reports complaints. Nevertheless, to describe change over time or the effect of treatment, a more elaborate questionnaire such as the FACT-Cog might be more suitable to be able to specify which sleep factors are most strongly associated with sr-NCF. The Pittsburgh Sleep Quality Index was the most frequently used measure of sleep. The PSQI could be recommended as a measure of sleep quality although, when necessary, it is recommended to add an objective measure for the quantity and quality of sleep, since patients’ reported sleep issues are often not confirmed by objective measurements [65].

This systematic review is the first one assessing the association between sleep and neurocognitive functioning in cancer survivors. One limitation of this review is that most studies consisted of a breast cancer population. This over-representation of breast cancer survivors makes the findings less generalizable to other cancer types, because of possible differences in treatment and the effect of the tumour on sleep and NCF. Another limitation is the exclusion of studies with cancer patients who are still undergoing treatment, leading to exclusion of (longitudinal) studies that had measurements pre- and post-cancer treatment. Both neurocognitive impairment and sleep issues are more often reported during, compared to after treatment, suggesting that a subgroup of patients recovers over time, while some survivors experience these issues long term.

In clinical practice, it is important that care providers are aware of the association between NCF and sleep and that treating poor sleep can be a way to decrease NCI in cancer survivors who report these issues.

The results of this review suggest that sleep and neurocognitive functioning are associated in cancer patients, with most evidence for an association with sr-NCF. Further research is necessary to be able to establish the effect of sleep interventions on sr-NCF, but particularly of oNCF.

### Supplementary Information

Below is the link to the electronic supplementary material.

Supplementary file1 (DOCX 20 KB)

## Data Availability

Not applicable.
