# Supplementary material for: Is poor sleep quality associated with poor neurocognitive outcome in cancer survivors? A systematic review
Source: J Cancer Surviv. 2022 May 2;18(2):207–22. doi: 10.1007/s11764-022-01213-z (PMC10960780; doi:10.1007/s11764-022-01213-z)
Supplement: Supplementary file 1 — Supplementary file1 (DOCX 20 KB) [file 11764_2022_1213_MOESM1_ESM.docx]

# Supplementary Information

**SI 1: Search strategy**

**Search strategy in PubMed (2021 May 26)**

| Concept | Query |
| --- | --- |
| #3: Sleep | "Sleep"[Mesh] OR sleep*[tiab] |
| AND | |
| #2: Neurocognitive Functioning | "Cognitive Dysfunction"[Mesh] OR "Cognition"[Mesh:noexp] OR "Cognition Disorders"[Mesh:noexp] OR cogniti*[tiab] |
| AND | |
| #1: Cancer | cancer[sb] |

**Search strategy in Embase.com (2021 May 26)**

| Concept | Query |
| --- | --- |
| #3: Sleep | 'sleep'/exp OR sleep*:ti,ab,kw |
| AND | |
| #2: Neurocognitive functioning | 'cognitive defect'/exp OR 'cognition'/de OR cogniti*:ti,ab,kw |
| AND | |
| #1: Cancer | 'neoplasm'/exp OR adenoma*:ti,ab,kw OR anticarcinogen*:ti,ab,kw OR blastoma*:ti,ab,kw OR cancer*:ti,ab,kw OR carcinogen*:ti,ab,kw OR carcinom*:ti,ab,kw OR carcinosarcoma*:ti,ab,kw OR chordoma*:ti,ab,kw OR germinoma*:ti,ab,kw OR gonadoblastoma*:ti,ab,kw OR hepatoblastoma*:ti,ab,kw OR ((hodgkin* NEXT/1 disease):ti,ab,kw) OR leukemi*:ti,ab,kw OR lymphangioma*:ti,ab,kw OR lymphangiomyoma*:ti,ab,kw OR lymphangiosarcoma*:ti,ab,kw OR lymphom*:ti,ab,kw OR malignan*:ti,ab,kw OR melanom*:ti,ab,kw OR meningioma*:ti,ab,kw OR mesenchymoma*:ti,ab,kw OR mesonephroma*:ti,ab,kw OR metasta*:ti,ab,kw OR neoplas*:ti,ab,kw OR neuroma*:ti,ab,kw OR nsclc:ti,ab,kw OR oncogen*:ti,ab,kw OR oncolog*:ti,ab,kw OR paraneoplastic:ti,ab,kw OR plasmacytoma*:ti,ab,kw OR precancerous:ti,ab,kw OR sarcoma*:ti,ab,kw OR teratocarcinoma*:ti,ab,kw OR teratoma*:ti,ab,kw OR tumor*:ti,ab,kw OR tumour*:ti,ab,kw |

**Search strategy in PsycINFO via EBSCO (2021 May 26)**

| # | Query |
| --- | --- |
| S3: Sleep | ( DE "Sleep" OR DE "Dreaming" OR DE "NREM Sleep" OR DE "Napping" OR DE "REM Sleep" OR DE "Sleep Onset" OR DE "Snoring" OR DE "Dream Content" OR DE "Dream Recall" OR DE "Lucid Dreaming" OR DE "Nightmares" OR DE "REM Dreams" ) OR TI sleep* OR AB sleep* OR KW sleep* |
| AND | |
| S2: Neurocognitive functioning | ( DE "Cognition" OR DE "Cognitive Impairment" ) OR TI cogniti* OR AB cogniti* OR KW cogniti* |
| AND | |
| S1: Cancer | DE "Neoplasms" OR DE "Benign Neoplasms" OR DE "Breast Neoplasms" OR DE "Endocrine Neoplasms" OR DE "Leukemias" OR DE "Nervous System Neoplasms" OR DE "Terminal Cancer" OR TI (adenoma* OR anticarcinogen* OR blastoma* OR cancer* OR carcinogen* OR carcinom* OR carcinosarcoma* OR chordoma* OR germinoma* OR gonadoblastoma* OR hepatoblastoma* OR hodgkin n3 disease OR leukemi* OR lymphangioma* OR lymphangiomyoma* OR lymphangiosarcoma* OR lymphom* OR malignan* OR melanom* OR meningioma* OR mesenchymoma* OR mesonephroma* OR metasta* OR neoplas* OR neuroma* OR nsclc OR oncogen* OR oncolog* OR paraneoplastic OR plasmacytoma* OR precancerous OR sarcoma* OR teratocarcinoma* OR teratoma* OR tumor* OR tumour*) OR AB (adenoma* OR anticarcinogen* OR blastoma* OR cancer* OR carcinogen* OR carcinom* OR carcinosarcoma* OR chordoma* OR germinoma* OR gonadoblastoma* OR hepatoblastoma* OR hodgkin n3 disease OR leukemi* OR lymphangioma* OR lymphangiomyoma* OR lymphangiosarcoma* OR lymphom* OR malignan* OR melanom* OR meningioma* OR mesenchymoma* OR mesonephroma* OR metasta* OR neoplas* OR neuroma* OR nsclc OR oncogen* OR oncolog* OR paraneoplastic OR plasmacytoma* OR precancerous OR sarcoma* OR teratocarcinoma* OR teratoma* OR tumor* OR tumour*) OR KW (adenoma* OR anticarcinogen* OR blastoma* OR cancer* OR carcinogen* OR carcinom* OR carcinosarcoma* OR chordoma* OR germinoma* OR gonadoblastoma* OR hepatoblastoma* OR hodgkin n3 disease OR leukemi* OR lymphangioma* OR lymphangiomyoma* OR lymphangiosarcoma* OR lymphom* OR malignan* OR melanom* OR meningioma* OR mesenchymoma* OR mesonephroma* OR metasta* OR neoplas* OR neuroma* OR nsclc OR oncogen* OR oncolog* OR paraneoplastic OR plasmacytoma* OR precancerous OR sarcoma* OR teratocarcinoma* OR teratoma* OR tumor* OR tumour*) |

**Search strategy in CINAHL via EBSCO (2021 May 26)**

| Concept | Query |
| --- | --- |
| S3: sleep | (MH "Sleep+") OR TI sleep* OR AB sleep* OR SU sleep* |
| AND | |
| S2: neurocognitive functioning | ( (MH "Cognition") OR (MH "Cognition Disorders") ) OR TI cogniti* OR AB cogniti* OR SU cogniti* |
| AND | |
| S1: cancer | (MH "Neoplasms+") OR TI (adenoma* OR anticarcinogen* OR blastoma* OR cancer* OR carcinogen* OR carcinom* OR carcinosarcoma* OR chordoma* OR germinoma* OR gonadoblastoma* OR hepatoblastoma* OR hodgkin n3 disease OR leukemi* OR lymphangioma* OR lymphangiomyoma* OR lymphangiosarcoma* OR lymphom* OR malignan* OR melanom* OR meningioma* OR mesenchymoma* OR mesonephroma* OR metasta* OR neoplas* OR neuroma* OR nsclc OR oncogen* OR oncolog* OR paraneoplastic OR plasmacytoma* OR precancerous OR sarcoma* OR teratocarcinoma* OR teratoma* OR tumor* OR tumour*) OR AB (adenoma* OR anticarcinogen* OR blastoma* OR cancer* OR carcinogen* OR carcinom* OR carcinosarcoma* OR chordoma* OR germinoma* OR gonadoblastoma* OR hepatoblastoma* OR hodgkin n3 disease OR leukemi* OR lymphangioma* OR lymphangiomyoma* OR lymphangiosarcoma* OR lymphom* OR malignan* OR melanom* OR meningioma* OR mesenchymoma* OR mesonephroma* OR metasta* OR neoplas* OR neuroma* OR nsclc OR oncogen* OR oncolog* OR paraneoplastic OR plasmacytoma* OR precancerous OR sarcoma* OR teratocarcinoma* OR teratoma* OR tumor* OR tumour*) OR SU (adenoma* OR anticarcinogen* OR blastoma* OR cancer* OR carcinogen* OR carcinom* OR carcinosarcoma* OR chordoma* OR germinoma* OR gonadoblastoma* OR hepatoblastoma* OR hodgkin n3 disease OR leukemi* OR lymphangioma* OR lymphangiomyoma* OR lymphangiosarcoma* OR lymphom* OR malignan* OR melanom* OR meningioma* OR mesenchymoma* OR mesonephroma* OR metasta* OR neoplas* OR neuroma* OR nsclc OR oncogen* OR oncolog* OR paraneoplastic OR plasmacytoma* OR precancerous OR sarcoma* OR teratocarcinoma* OR teratoma* OR tumor* OR tumour*) |

**SI 2: Description of the AXIS tool**

*Introduction*

1 Were the aims/objectives of the study clear?

*Methods*

2 Was the study design appropriate for the stated aim(s)?

3 Was the sample size justified?

4 Was the target/reference population clearly defined? (Is it clear who the research was about?)

5 Was the sample frame taken from an appropriate population base so that it closely represented the target/reference population under investigation?

6 Was the selection process likely to select subjects/participants that were representative of the target/reference population under investigation?

7 Were measures undertaken to address and categorise non-responders?

8 Were the risk factor and outcome variables measured appropriate to the aims of the study?

9 Were the risk factor and outcome variables measured correctly using instruments/ measurements that had been trialled, piloted or published previously?

10 Is it clear what was used to determined statistical significance and/or precision estimates? (eg, p values, CIs)

11 Were the methods (including statistical methods) sufficiently described to enable them to be repeated?

*Results*

12 Were the basic data adequately described?

13 Does the response rate raise concerns about non-response bias?

14 If appropriate, was information about non-responders described?

15 Were the results internally consistent?

16 Were the results for the analyses described in the methods, presented? Discussion

17 Were the authors’ discussions and conclusions justified by the results?

18 Were the limitations of the study discussed?

*Other*

19 Were there any funding sources or conflicts of interest that may affect the authors’ interpretation of the results?

20 Was ethical approval or consent of participants attained?

Downes, M. J., et al. (2016). "Development of a critical appraisal tool to assess the quality of cross-sectional studies (AXIS)." BMJ Open **6**(12): e011458.

**SI 3: AXIS tool scores**

|  | Caplette-Gingras 2013 | Ehlers 2018 | Ehlers 2020 | Hartman 2015 | Henneghan 2018 | Hutchinson 2021 | Jean-Pierre 2015 | Jung 2017 | Liou 2019 | Syarif 2019 | Van Dyk 2018 | Von Ah 2015 |
| --- | --- | --- | --- | --- | --- | --- | --- | --- | --- | --- | --- | --- |
| score | **17** | **16** | **15** | **15** | **16** | **15** | **15** | **16** | **17** | **16** | **16** | **16** |

Risk of bias assessment for the cross-sectional studies using the AXIS tool (score out of a total score of 19. A higher score indicates a lower risk of bias.
